# Malarial Retinopathy: the Summary on Contemporaneous Hypothesis

**Published:** 2012

**Authors:** Viroj Wiwanitkit

**Affiliations:** Adjunct professor, Joseph Ayobabalola University, Nigeria, Visiting Professor, Hainan Medical University, China

**Keywords:** Retinopathy, Malaria, Pathophysiology

## Abstract

Malaria is a life-threatening disease caused by parasites that are transmitted to people through the bites of infected mosquitoes. Malaria retinopathy is misdiagnosed in the clinical setting, leading to a failure to treat other life-threatening illnesses. Indeed, the problem can be severe and should be the focus in tropical ophthalmology. In this brief article, the author summarises and comments on the present hypothesis for malarial retinopathy. This hypothesis could be justified by further basic and clinical studies.

## INTRODUCTION

Malaria is one of the most important mosquito-borne infectious diseases. This tropical infection is still the present public health threat of the world. Annually, thousands of malarial cases are reported from around the world, and many death cases can be seen. In general, the complications of malaria can be expected and this is the primary issue in malarial management. Several organs can be affected in complicated malaria, and the eye can also be affected. The ocular manifestations of malaria are an interesting issue in ophthalmology [1]. The problem of the retina is that malaria can be seen but is usually forgotten. Indeed, the problem can be severe and should be the focus in tropical ophthalmology. In a recent report from Africa, it was found that retinopathy was not rare and there are many forms of retinopathy including “retinal whitening, haemorrhages, unique vessel abnormalities, papilloedema, and cotton wool” appearances [2]. It is also noted that ophthalmoscopy can be helpful in managing cases of severe malaria as a useful tool for the early diagnosis of cerebral malaria [3]. Beare et al. concluded that “Studies of the retina and retinal blood vessels provide an unparalleled opportunity to visualise an infected microvasculature and its effect on neural tissue *in vivo*” [4]. In this article, the author briefly summarises and comments on the present hypothesis for malarial retinopathy.

## HYPOTHESES

**Relative hypoxia and reversible intracellular oedema**

In malarial infection, the primary affected cells are the erythrocytes. Hence, it is not surprising that the loss of function of red blood cells can be expected. An important function of red blood cell is carrying oxygen, so the disturbance of oxygenation in malarial patients can be expected. Sequestered erythrocytes infected by *Plasmodium falciparum* are common, which could be the explanation for the hypoxic insult [5]. Vessel occlusion and filling defects might be important problematic outcomes of the sequestration of infected erythrocytes [6-7]. According to a recent report by Beare et al., it was found that the retina problems in malarial patients were not related to the visual acuity loss [5]. The reversible retinal pathology was also observed [5]. Indeed, ischaemia due to hypoxic insult is an important pathological state that can be seen in both the retina and eye of patients with severe malaria [8]. The ERG study also confirmed the ischaemic pattern of the retina in cases of malarial retinopathy [9]. The significant observed pattern of ERG is a cone b wave function [9]. 

**Nutritional deficiency as underlying factor for malarial retinopathy**

Some scientists do not agree with the concept of hypoxia and occlusion due to sequestrated red cells. Hero et al. observed that the “blood-retina barrier and retinal vascular flow remain substantially normal despite widespread pathological features” . This is against the hypothesis of hypoxic insult. Hero et al. proposed that nutritional deficiency should be the cause of retinal impairment in malarial retinopathy [10]. The roles of some nutritional problems are mentioned due to their relationships with malarial retinopathy. Folate deficiency is mentioned as a cause of retinal haemorrhage in severe malaria [11]. It is reported that malarial cases with anaemia have a higher chance to develop malarial retinopathy [12]. Also, Lewallen et al. reported that malarial retinopathy “is associated with low serum vitamin A levels” [12]. The underlying vitamin A deficiency-related retinal problems are thought to be a factor that aggravates the severity of malarial retinopathy [13].

## CONCLUSION

At present, there are many hypotheses that explain the pathophysiology of malarial retinopathy. The supportive evidences for each theory support the proposed hypothesis. The exact pathophysiology still needs further studies for verification. The exact process might be complex and based on a number of hypotheses.

## DISCLOSURE

The authors report no conflicts of interest in this work.

## References

[1] Gabay JE, Mayers M (1997). Ocular manifestations of systemic infections. Curr Opin Ophthalmol.

[2] Lewallen S, Harding SP, Ajewole J, Schulenburg WE, Molyneux ME, Marsh K, Usen S, White NJ, Taylor TE (1999). A review of the spectrum of clinical ocular fundus findings in P. falciparum malaria in African children with a proposed classification and grading system. Trans R Soc Trop Med Hyg.

[3] Hirneiss C, Klauss V, Wilke M, Kampik A, Taylor T, Lewallen S (2005). [Ocular changes in tropical malaria with cerebral involvement--results from the Blantyre Malaria Project]. Klin Monbl Augenheilkd.

[4] Beare NA, Taylor TE, Harding SP, Lewallen S, Molyneux ME (2006). Malarial retinopathy: a newly established diagnostic sign in severe malaria. Am J Trop Med Hyg.

[5] Beare NA, Southern C, Kayira K, Taylor TE, Harding SP (2004). Visual outcomes in children in Malawi following retinopathy of severe malaria. Br J Ophthalmol.

[6] Maude RJ, Dondorp AM, Abu Sayeed A, Day NP, White NJ, Beare NA (2009). The eye in cerebral malaria: what can it teach us?. Trans R Soc Trop Med Hyg.

[7] Beare NA, Harding SP, Taylor TE, Lewallen S, Molyneux ME (2009). Perfusion abnormalities in children with cerebral malaria and malarial retinopathy. J Infect Dis.

[8] Essuman VA, Ntim-Amponsah CT, Astrup BS, Adjei GO, Kurtzhals JA, Ndanu TA, Goka B (2010). Retinopathy in severe malaria in Ghanaian children--overlap between fundus changes in cerebral and non-cerebral malaria. Malar J.

[9] Lochhead J, Movaffaghy A, Falsini B, Harding S, Riva C, Molyneux M (2010). The effects of hypoxia on the ERG in paediatric cerebral malaria. Eye (Lond).

[10] Hero M, Harding SP, Riva CE, Winstanley PA, Peshu N, Marsh K (1997). Photographic and angiographic characterization of the retina of Kenyan children with severe malaria. Arch Ophthalmol.

[11] Eisenhut M (2007). Role of folate deficiency in the pathogenesis of retinal and cerebral hemorrhages in cerebral malaria. Am J Trop Med Hyg.

[12] Beare NA, Southern C, Chalira C, Taylor TE, Molyneux ME, Harding SP (2004). Prognostic significance and course of retinopathy in children with severe malaria. Arch Ophthalmol.

[13] Lewallen S, Taylor TE, Molyneux ME, Semba RD, Wills BA, Courtright P (1998). Association between measures of vitamin A and the ocular fundus findings in cerebral malaria. Arch Ophthalmol.

